# Conditioned media from human umbilical cord blood-derived mesenchymal stem cells stimulate rejuvenation function in human skin

**DOI:** 10.1016/j.bbrep.2018.10.007

**Published:** 2018-10-25

**Authors:** Yoon-Jin Kim, Dong Hee Seo, Seung Hee Lee, Sung-Hoon Lee, Geun-Ho An, Hee-Jin Ahn, Daekee Kwon, Kwang-Won Seo, Kyung-Sun Kang

**Affiliations:** a Stem Cells and Regenerative Bioengineering Institute, Kangstem Biotech Co., Ltd., 2nd floor, Biotechnology center, #81 Seoul National University, 1 Gwanak-ro, Gwanak-gu, Seoul 08826, Republic of Korea; b Adult Stem Cell Research Center, College of Veterinary Medicine, Seoul National University, 1 Gwanak-ro, Gwanak-gu, Seoul 08826, Republic of Korea

**Keywords:** Conditioned media, Cosmetics, Mesenchymal stem cells, Skin, Umbilical cord blood-derived mesenchymal stem cells conditioned media (USC-CM)

## Abstract

Developing treatments that inhibit skin aging is an important research project. Rejuvenation, which focuses on prevention of skin aging, is one of the major issues. Recent studies suggested that mesenchymal stem cells (MSCs) secrete many cytokines, which are important in wound healing. In this study, we investigated the effect of human umbilical cord blood-derived mesenchymal stem cells conditioned media (USC-CM) in cutaneous wound healing and collagen synthesis. We found that USC-CM has many useful growth factors associated with skin rejuvenation, such as Epithelial Growth Factor (EGF), basic Fibroblast Growth Factor (bFGF), Platelet Derived Growth Factor (PDGF), Hepatocyte Growth Factor (HGF), Collagen type 1, and especially, one of the rejuvenation factors, the growth differentiation factor-11 (GDF-11). Our *in vitro* results showed that USC-CM stimulate growth and extracellular matrix (ECM) production of Human Dermal Fibroblasts (HDFs) compared to those of other MSCs conditioned media (CM) from different origins. Moreover, we evaluated the roles of GDF-11. The results showed that GDF-11 accelerates growth, migration and ECM production of HDFs. Our *In vivo* results showed that topical treatment of USC-CM showed anti-wrinkle effect and significantly increased dermal density in women. In conclusion, USC-CM has various useful growth factors including GDF-11 that can stimulate skin rejuvenation by increasing growth and ECM production of HDFs.

## Highlights

• USC-CM has various growth factors associated with skin rejuvenation including GDF-11 that strongly promoted HDFs migration, collagen synthesis *in vitro* compared with HDF- and AD-MSC-CM.
• USC-CM increased dermal density and decreased skin wrinkle in human.

## Graphical abstract

Effect of USC-CM including GDF-11 *in vivo* and *in vitro*.

**Figure:**
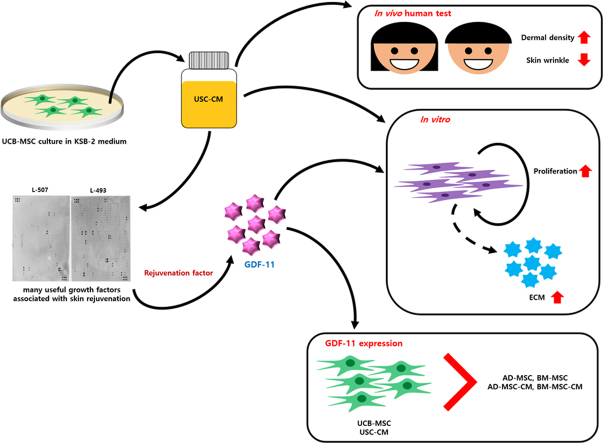

## 1 Introduction

Skin rejuvenation has become the focus of cosmeceuticals, in which various medical treatments and products are used for anti-aging. Skin aging can be divided into two different types of age-dependent aging (intrinsic aging) and photo-aging (extrinsic aging) owing to the physiological and environmental factors [1], [2], [3]. Impairment of skin integrity is one of the most prominent indications of skin aging. In age-dependent aging the main indication of aging is the appearance of wrinkles on skin, while in photo-aging the indications of aging are accompanied with various symptoms such as wrinkles, loss of elasticity, discoloration, hyperkeratosis, irregular pigmentation, and other various neoplasms [4], [5].

The skin extracellular matrix (ECM) that consists of glycosaminoglycans, collagen and elastin, is crucial for skin morphology and functions such as growth and elasticity [6]. The degradation of ECM that occurs along with skin aging is related to the increase of the activity of enzymes such as hyaluronidase, elastase and collagenase [7]. Photo-aging causes production of collagenase by Human Dermal Fibroblasts (HDFs), which degenerates collagen production and is exposed on skin as wrinkles. The collagen accounts for up to 70% of the weight of the dermis. The major collagens are type 1 and 3 that are largely responsible for the tensile strength of the dermis and minimization of the wrinkles. Elastin is a major protein component of tissues that helps skin to return to its original position when it is poked. It provides natural elasticity and strength and plays an important role in tissue reparation of the human body [8]. It is noteworthy that the strength and resiliency of skin are governed by proper and uniform arrangement of collagen (both type 1 and 3) fibrils and elastin in the dermis [9].

Mesenchymal stem cells (MSCs) are multipotent cells derived from a variety of tissues including bone marrow, adipose tissues and umbilical cord blood. Primitive umbilical cord blood-derived mesenchymal stem cells (UCB-MSCs) have biological advantages, compared to other MSCs [10], [11], [12]. MSCs are the most important cells in the skin, as they are the source for continuous regeneration of the epidermis [13]. Recent studies suggested that MSCs stimulated HDFs *via* paracrine effects and enhanced cutaneous wound healing [14], [15].

MSCs secrete many cytokines and growth factors such as Epidermal growth factor (EGF), basic Fibroblast growth factor (bFGF), Transforming growth factor-beta (TGF-b), which are important in cell growth and maintaining skin tissues [16], [17]. However, it is still unclear what their beneficial roles in growth factors for skin rejuvenation [18].

Growth differentiation factor-11 (GDF-11) is a member of TGF-b superfamily and a secreted signal that acts globally to specify positional identity along the anterior/posterior axis of vertebrates [19]. GDF-11, also known as bone morphogenetic protein-11, is considered as a rejuvenation factor in symbiotic animal experiment [20]. When young and old animals have a common blood circulation, the elder animal becomes younger in many aspects since soluble factors from blood of young animals can affect the elder animals. Katsimpardi et al. claimed GDF-11 in the serum from young animal is a pivotal soluble factor for rejuvenation [21]. The relationship between GDF family and skin rejuvenation or skin ECM has not been proven before. The only known thing is that GDF-5, a member of GDF family, influences multiple tissues composed primarily of Collagen type 1, with consistent biomechanical effects on non-weight-bearing tissues such as tail tendon and skin [22]. In this study, we hypothesized that young blood-originated hMSCs could produce rejuvenating factors that can attenuate the aging of human skins with various secreted soluble factors.

## 2 Materials and methods

### 2.1 Culture of AD-MSC, BM-MSC and UCB-MSC

UCB-MSCs were isolated from Human umbilical cord bloods approved by the FORMIZ WOMEN's Hospital (IRB No. 219255-201305-BR-001, Seoul, Korea) with previously described method [23]. Human adipose tissue samples were acquired from the KODI MEDICAL (Seoul, Korea, IRB No. 219255-201407-BR-001-01). Human bone marrow-derived mesenchymal stem cells were acquired from the SEVERANCE HOSPITAL (Seoul, Korea, IRB No. 4–2008–0643). AD-MSC, BM-MSC and UCB-MSC were cultured and expanded up to passage 5 at 37 ℃ and 5% CO_2_ in KSB-3 (Irvine scientific, Santa Ana, CA) with 10% fetal bovine serum (FBS) (Gibco) and characterized it as previously reported [24], [25].

### 2.2 Preparation of HDF-CM, AD-MSC-CM, BM-MSC-CM and USC-CM

HDF, AD-MSC, BM-MSC and UCB-MSCs (1.98 × 10^5^ cells/Flask) were seeded in T-25 flask and cultured for 48 h in KSB-3 (Irvine Scientific, California) with 10% FBS. After PBS washing twice, the culture medium was changed to KSB-2 media; DMEM (Gibco) containing EGF (10 ng/ml) and bFGF (10 ng/ml), followed by incubation period of 96 h. Conditioned media (CM) of MSCs and HDFs were collected, centrifuged at 1500 rpm for 5 min, and finally filtered using a 0.22 µm syringe filter. The conditioned media were measured with GDF-11 ELISA kit (R&D systems, Minneapolis, MN) according to the manufacturer's protocol.

### 2.3 Human antibody array

Human proteins analyzed by using a Human Antibody Array 1000 (Cat. No. AAH-BLM-1000-4, RayBiotech) according to the manufacturer's instructions. Membranes were developed using detection buffer and quantified using a densitometer. After developing, films were scanned and the images processed and quantified using Image J software (National Institutes of Health). Signal intensity was normalized to internal positive controls for comparison.

### 2.4 Proliferation assay

HDFs (1 × 10^3^ cells/well) were seeded in 96-well plates and cultured for 24 h in KSB-3 medium. After washing, the medium was replaced by control medium (KSB-2 media) or varying conditioned medium (HDF-CM, AD-MSC-CM and USC-CM). To measurement of HDFs proliferation with GDF-11, the medium was replaced by control medium (DMEM) or various concentrations of GDF-11 (0.1 μg/ml, 0.2 μg/ml). After 72 h, HDFs proliferation was measured using a CCK-8 kit (Dojindo, Gaithersburd, USA). HDFs were added to 10 μl of the CCK-8 solution, and incubated for 3 h, and then the absorbance was measured at 450 nm using a microplate reader (Tecan, Mannedorf, Switzerland). Optical density values from each well were used to calculate the relative cell numbers by comparing to the standard curves. The protein content of HDFs was determined by using a DC™ protein assay kit (Bio-Rad, Philadelphia, USA).

### 2.5 Scratch assay

7 × 10^5^ HDFs were seeded into the cell culture system by using the ibidi Culture-Insert (No. 81176, ibidi GmbH, Munich, Germany,). This approach provides two cell culture reservoirs with a separation wall of 500 µm thick. For the measurement of cell migration, the silicon inserts were removed after 24 h. The gaps created were washed and each well was filled with control medium (KSB-2 media or DMEM) or varying conditioned medium (HDF-CM, AD-MSC-CM and USC-CM) as well as Human GDF-11 (R&D systems, Minneapolis, USA). HDFs were photographed 72 h after wounding by phase-contrast microscopy and measured manually with Image J analysis. The data were reported as the ratio of migration relative to the control.

### 2.6 Co-culture of HDFs with HDF-CM, AD-MSC-CM and USC-CM

HDFs (2 × 10^5^ cells/well) were seeded in 6-well plates and cultured for 24 h in KSB-3 medium. After washing, each well was filled with control medium (KSB-2 media) or varying conditioned medium (HDF-CM, AD-MSC-CM and USC-CM), followed by incubation period of 24 h.

### 2.7 Co-culture of HDFs with GDF-11

HDFs (2 ×10^5^ cells/well) were seeded in 6-well plates and cultured for 24 h in KSB-3 medium. After washing, different concentrations of GDF-11 (0.01 μg/ml, 0.1 μg/ml) with serum-free culture medium (DMEM) were changed for additional 24 h culture.

### 2.8 Reverse transcription polymerase chain reaction (RT-PCR) and real-time PCR

Total cellular RNA was extracted using an RNA mini kit (Invitrogen, Waltham, USA), followed by a reverse transcription using a cDNA synthesis kit (Bioneer, Daejeon, Korea). cDNA was amplified using the Accupower PCR premix (Bioneer) or followed by quantitative PCR using the SYBR Green PCR Master Mix (Applied Biosystems, Foster City, CA) with each primer. Each gene expression level was normalized with GAPDH or RPL13A as housekeeping controls. The primer sequences were listed in Supplementary Table 1.

### 2.9 Western blot analysis

Cells were collected, washed, and extracted with PRO-PREP™ protein extraction solution (iNtRON Biotech, Sungnam, Korea). Fifty micrograms of proteins were separated on an 8% sodium dodecyl sulfate-polyacryamide gel by electrophoresis. The proteins were transferred to Nitrocellulose membranes (Bio-rad, Hercules, CA). The membranes were incubated with antibodies of Collagen type 1, Collagen type 4, Fibronectin, Elastin, TGFb Receptor1, SMAD2, p-SMAD2, SMAD3, p-SMAD3 and GAPDH (1:1000; abcam, Cambridge, USA). Then, the membranes were washed and incubated with a secondary antibody conjugated to horseradish peroxidase (1:5000, abcam). Membranes were developed using ECL (GE Healthcare, Pittsburgh, USA) and quantified using a densitometer. Mean pixel density was quantified using Image J analysis Representative western blot of Collagen type 1, Collagen type 4, Fibronectin, Elastin, TGFb Receptor1, SMAD2, p-SMAD2, SMAD3 and p-SMAD3 those of either total protein or GAPDH to correct for protein loading in the case of cellular lysate extracts.

### 2.10 *In vivo* human test

*In vivo* tests on human were performed as described in the Supplementary Materials and Methods Section.

### 2.11 Statistical analysis

Data are representatives of three independent experiments. Statistical analysis was performed with SPSS version 17.0 (SPSS Inc., Chicago, IL, USA). Normality was tested with Shapiro-Wilk test. Homogeneity between groups at baseline was tested by ANOVA. Differences intergroup were calculated by student's T test, ANOVA, and ANOVA with a post hoc analysis using the Dunnett's test. Statistical significance was considered when *P* < 0.05, 0.01 and 0.001.

## 3 Results

### 3.1 UCB-MSC secretes cytokines that stimulate HDFs growth

**Fig. 1:**
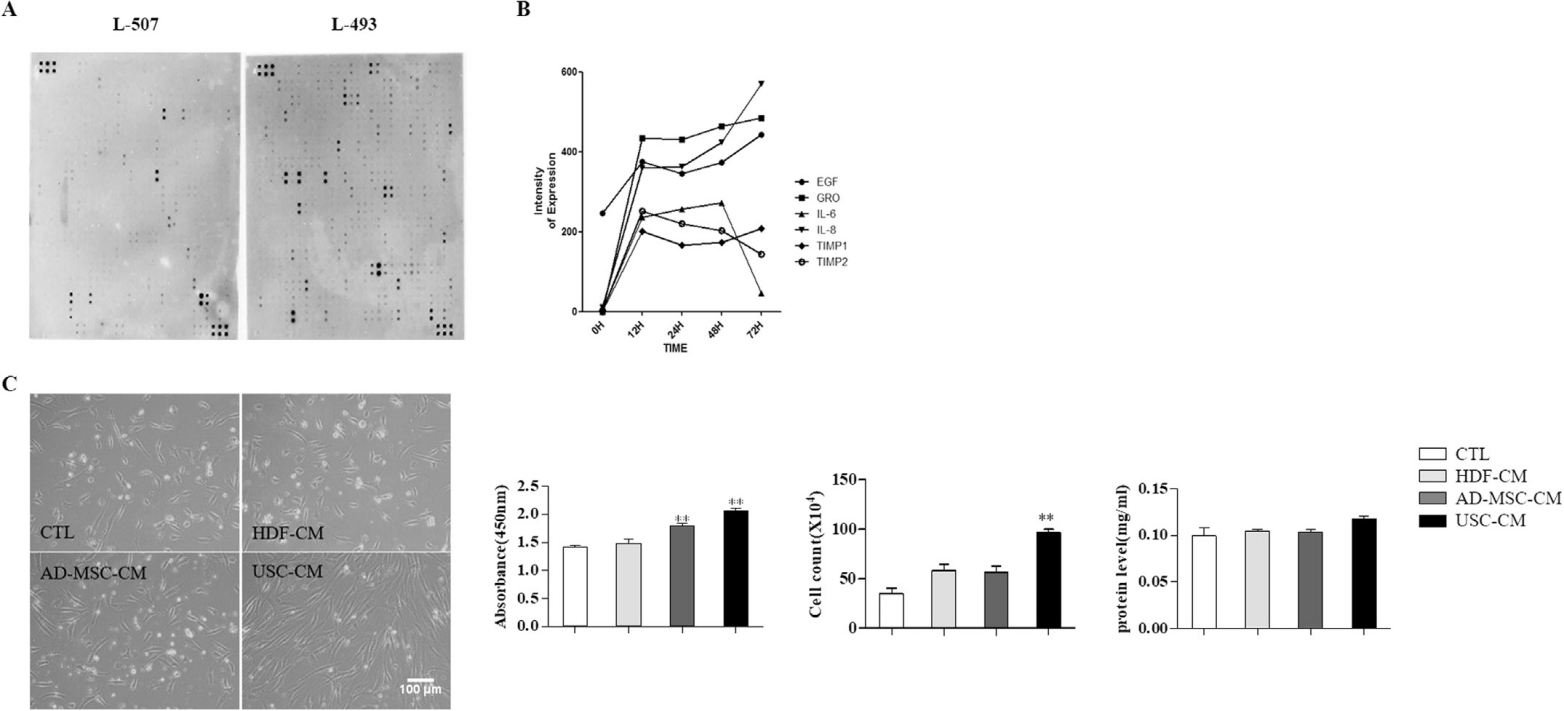
Selection of conditioned media (CM). (A) Human antibody array analysis of USC-CM. (B) Time line of cytokine expression of USC-CM. (C) HDFs growth and total protein content after various CM (CTL; KSB-2 media, HDF-CM, AD-MSC-CM and USC-CM) (x100). Data are represented as the mean ± SEM. ^**^P < 0.01.

For the anti-aging effect in human skin, HDFs growth and its production of ECM are the most essential factors. Therefore, we focused on the HDFs growth and the ECM production. UCB-MSC secrete various useful cytokines (Fig. 1A). They secrete several different cytokines, which were detected with cytokine dot-blot (Fig. 1A). In addition, we compared UCB-MSC conditioned media (USC-CM) with two different MSC-CM (AD-MSC-CM, BM-MSC-CM) using the human growth factor antibody array C1 (Cat. No. AAH-GF-1–8, RayBiotech) (Supplementary Fig. 1). USC-CM strongly contained skin-related proteins compared with AD- and BM-MSC-CM. Most of the major cytokines were secreted from UCB-MSC within 48 h (Fig. 1B). Four days of culture with KSB-2 media was evaluated as a proper culture method for making conditioned media. In this USC-CM, major growth factors were evaluated with ELISA (Supplementary Table 2). EGF, bFGF, HGF, PDGF and Collagen type 1 were highly increased in USC-CM. Additionally, HDFs and AD-MSC were cultured with KSB-2 media with the same culture method used for UCB-MSC. Four days later, each type of CM from UCB-MSC, AD-MSC and HDFs was collected and added to HDFs instead of the regular HDFs culture medium. After 72 h of culture in these conditioned media, we found that HDFs showed greater proliferation in USC-CM than in AD-MSC-CM and HDF-CM. There were no significant effects of total protein contents on HDFs (Fig. 1C). Therefore, we selected USC-CM, which was cultured with UCB-MSC for 4 days in KSB-2 media for the following experiments.

### 3.2 USC-CM promoted HDFs migration and ECM production *in vitro*

**Fig. 2:**
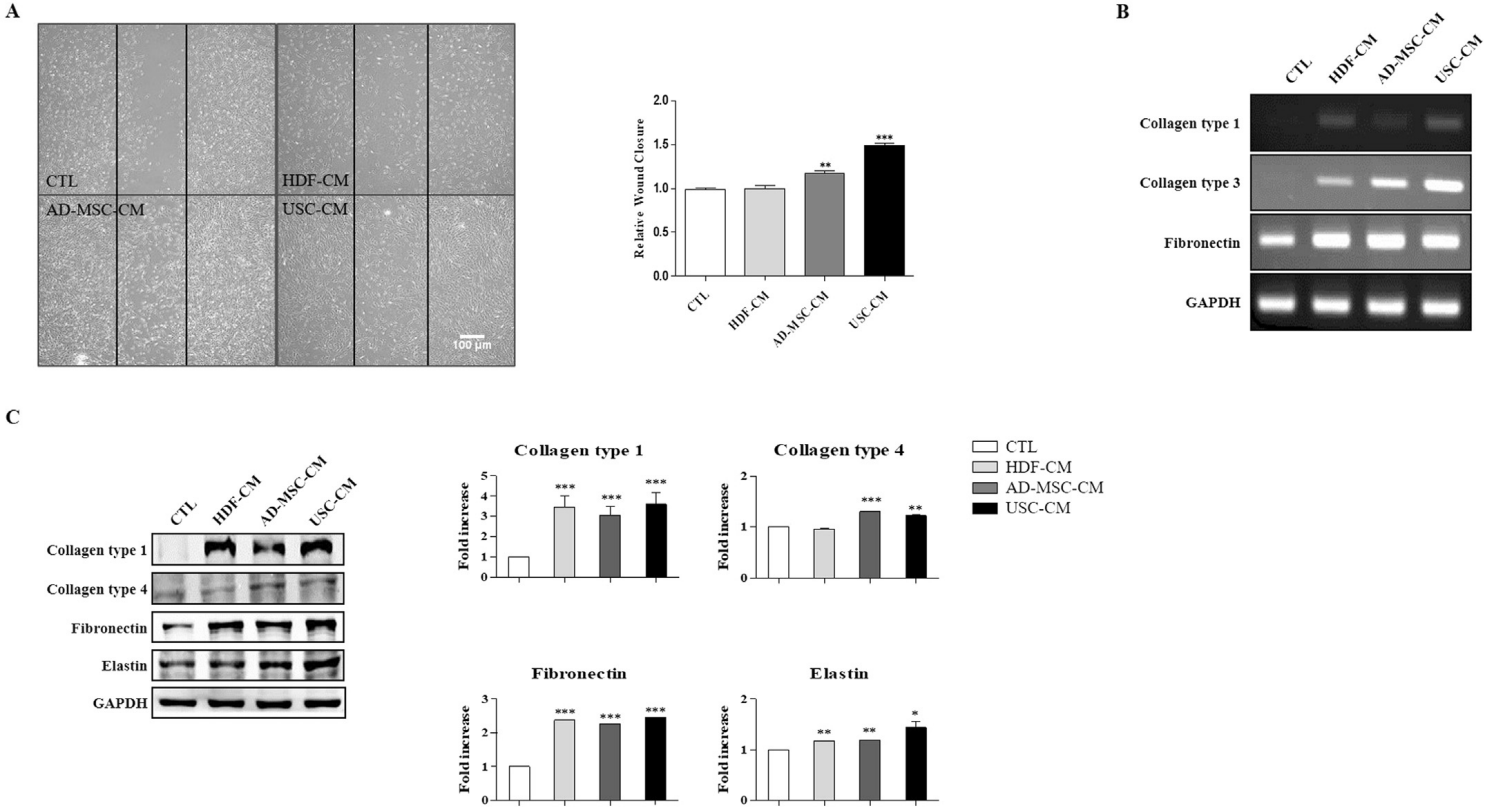
USC-CM promoted HDFs migration and ECM production *in vitro*. (A) Migration of HDFs after CM culture. USC-CM is most effective in HDFs migration. (x40) (B) ECM gene expression of HDFs treated with each CM (CTL; KSB-2 media, HDF-CM, AD-MSC-CM and USC-CM). (C) ECM secretion of HDFs treated with each CM. Data are represented as the mean ± SEM. ^*^P < 0.05, ^**^P < 0.01 and ^***^P < 0.001.

In order to determine HDFs proliferation with various conditioned media, scratch assay was performed (Fig. 2A). USC-CM showed the most distinguished proliferation and recovery/migration property compared to those of HDF-CM and AD-MSC-CM. Therefore, we evaluated the ECM gene expression of HDFs treated with each type of CM. The results of Collagen type 1 and 3 gene expression were significantly increased (Fig. 2B). These results were confirmed in protein levels with western blot analysis (Fig. 2C). Especially, from USC-CM culture Collagen type 1 and Elastin were most abundantly secreted from HDFs compared to HDF-CM and AD-MSC-CM. All data demonstrate that USC-CM strongly promoted HDFs migration, collagen synthesis *in vitro* compared with HDF- and AD-MSC-CM.

### 3.3 UCB-MSC expressed and secreted high level of GDF-11

**Fig. 3:**
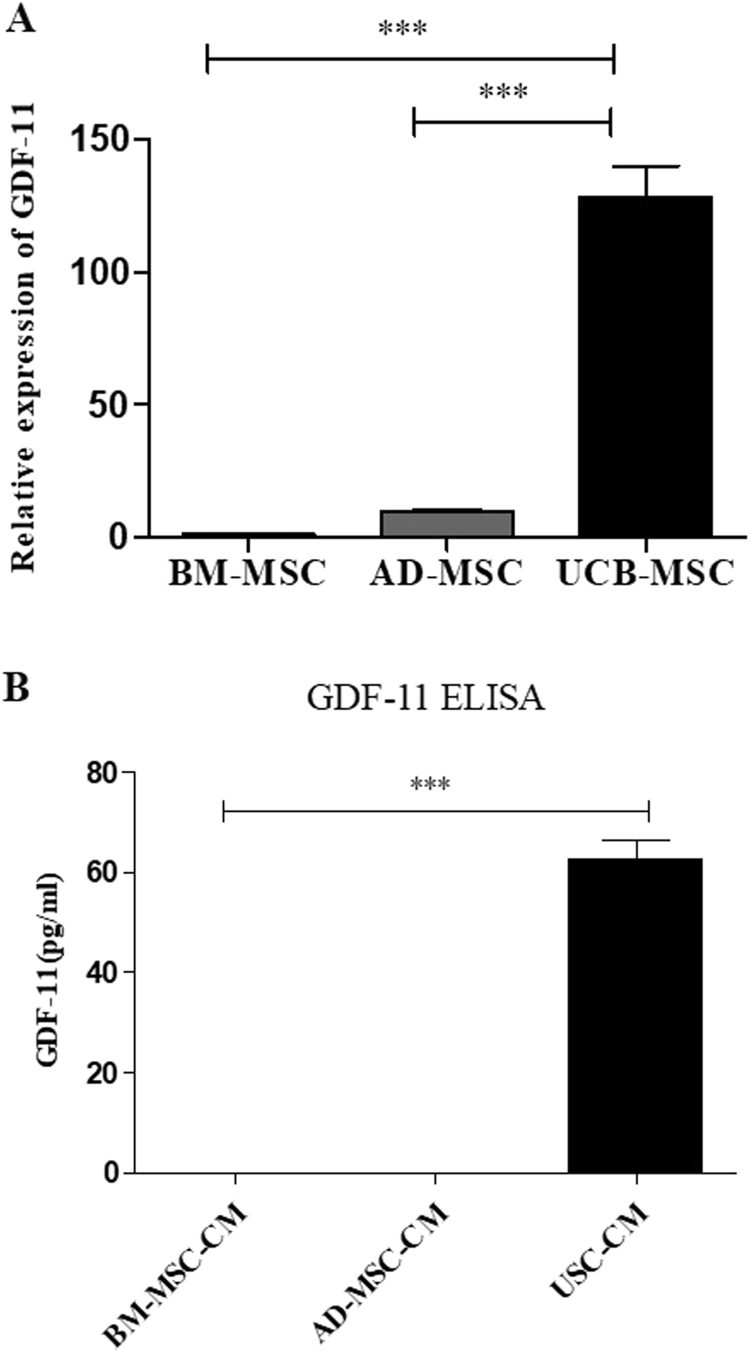
GDF-11 expression of hMSCs. (A) Gene expression of GDF-11 in hMSCs with each culture condition. (B) The level of production GDF-11 was measured using ELISA kit. Data are represented as the mean ± SEM. ^**^P < 0.01 and ^***^P < 0.001.

Among secreted cytokines from UCB-MSC, one of the known rejuvenation factors, GDF-11, was found (Supplementary Table 2). The GDF-11 expression was compared among the three types of conventionally used human mesenchymal stem cells (hMSCs), BM-MSC, AD-MSC and UCB-MSC. Among these three types of hMSCs, UCB-MSC showed the most abundant RNA expression of GDF-11 as expected since UCB-MSC was originated from the youngest tissues (Fig. 3A). The RNA expression level of GDF-11 was more than 100 folds greater in UCB-MSC than BM-MSC and AD-MSC. Each of the hMSCs was cultured in KSB-2 media for 4 days to compare the GDF-11 protein expression levels among the three clones of hMSCs (Fig. 3B). GDF-11 protein secretion from UCB-MSC was also the highest among the 3 different hMSCs clones. This analysis demonstrated that GDF-11 expression/secretion was the highest in UCB-MSC compared to those of other hMSCs.

### 3.4 GDF-11 promoted HDFs migration and ECM production *in vitro*

**Fig. 4:**
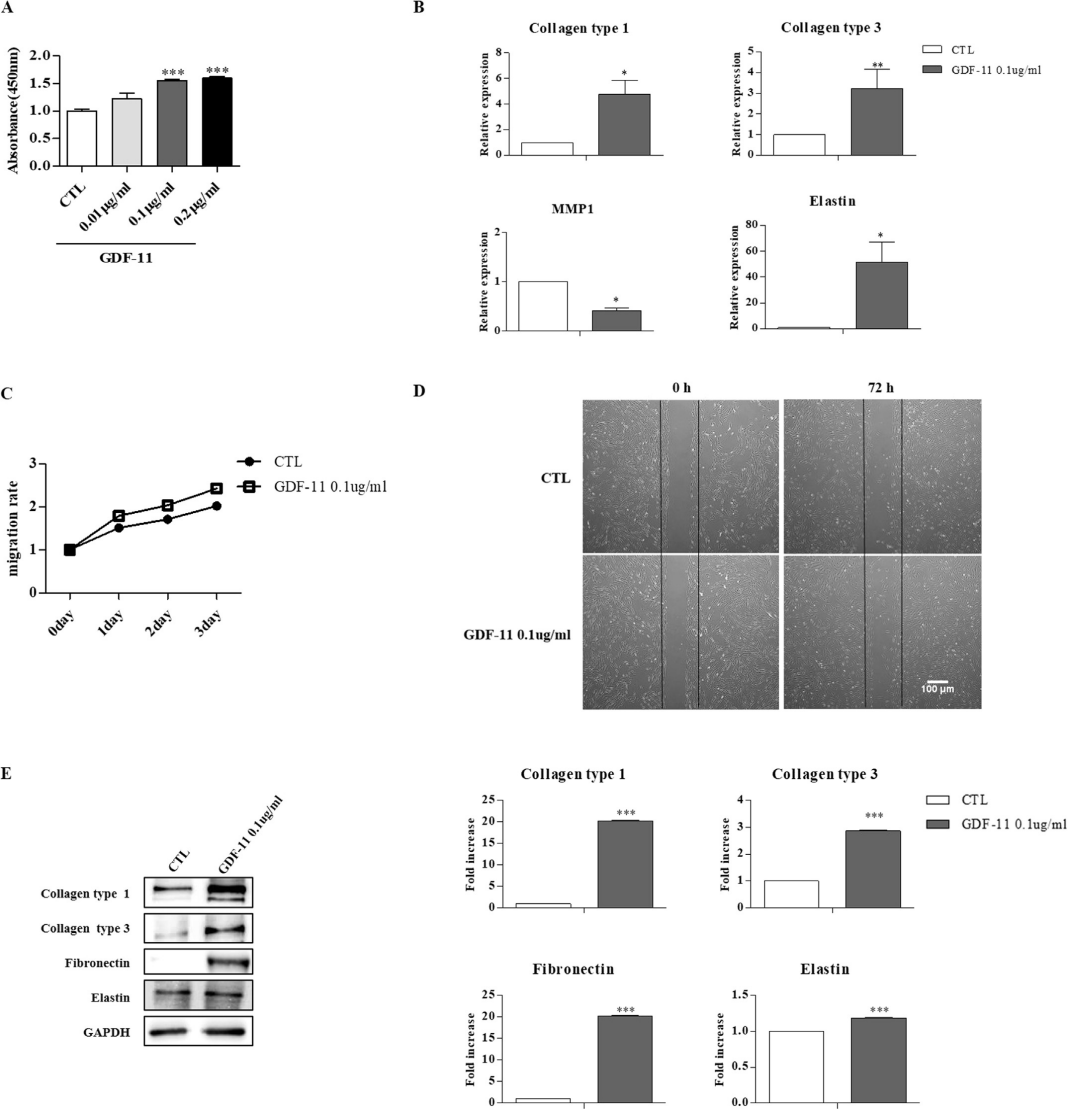
GDF-11 promoted HDFs migration and ECM production *in vitro*. (A) The proliferation of HDFs treated GDF-11 (0.01 μg/ml, 0.1 μg ml) treatment. (B) qRT-PCR analysis for ECM expression HDFs threated with GDF-11. (C) Time-course growth rate of HDFs following treated with GDF-11. (D) Scratch assay of HDFs following treated with GDF-11 for 72 h. (E) Western blot analysis for ECM secretion of HDFs threated with GDF-11. Data are represented as the mean ± SEM. ^*^P < 0.05, ^**^P < 0.01 and ^***^P < 0.001.

UCB-MSC secretes various cytokines and can stimulate ECM production. Among these cytokines, GDF-11 was proven to be an essential factor for rejuvenation. To verify the role of GDF-11 in HDFs, we evaluated the cell growth and ECM gene expression of HDFs treated with GDF-11. We investigated HDFs growth ability of GDF-11 (0.01 μg/ml, 0.1 μg/ml). GDF-11 showed accelerated proliferation of HDFs and showed dose-dependent manner (Fig. 4A). Therefore, the optimal concentration of GDF-11 was decided as 0.1 μg/ml. In addition, the results of Collagen type 1, 3 and Elastin gene expression were significantly increased. Furthermore, one of collagenase, MMP-1 expression was significantly decreased in GDF-11 (0.1 μg/ml) compared to control group (Fig. 4B). Consecutively, the proliferation of HDFs were significantly increased in GDF-11 (0.1 μg/ml) group compared to control group for 72 h (Fig. 4C). To determine the effect of GDF-11 (0.1 μg/ml) on migration of HDFs, scratch assay was performed. Images of the closing area at 0 and 72 h is shown in Fig. 4D. These expressions and secretions were confirmed in protein expression levels, which were detected in western blot analysis. The protein levels of Collagen type 1, 3, Fibronectin and Elastin were significantly increased in GDF-11 group compared to control group (Fig. 4E). All data demonstrate that GDF-11 promoted HDFs migration, collagen synthesis *in vitro.*

### 3.5 USC-CM contained cosmetics increased dermal density and decreased skin wrinkle in human

For *in vivo* test, 22 volunteers (18–55 years-old women) were selected. After IRB and signature of consent, 10% cryo-preserved USC-CM in cream base were treated daily to their skin. The subjects were observed every 2 weeks and the final observation was performed at 4 weeks from the starting point (Supplementary Fig. 2). In this *in vivo* test, there was no irritation, stinging or any adverse reaction observed. Dermal density was measured with ultrasound and it was increased as following treatment times (Supplementary Fig. 2A). The skin density at 4 weeks after the treatment was significantly increased by 2.46% when compared to that of before treatment (Supplementary Table 3). The skin wrinkles of eye-end area were also decreased after the treatment (Supplementary Fig. 2B). These results were measured and counted with digital micro mirror devices. Four weeks after the treatment, skin wrinkles of eye-end area were significantly decreased (Supplementary Table 4). Especially, maximum of all peak-to-valley value (Rmax) and maxium profile peak-height (Rp) were significantly decreased.

## 4 Discussion

Histologically, photo-aged skin shows marked alterations in ECM composition. HDFs play key roles in these changes because they are the source of extracellular matrix proteins and adhesive molecules [26]. Stem cells have self-renewing and differentiation potentials. According to their benefits, stem cell therapy has prevent a disease and reparation of tissue. Human MSCs secrete various growth factors, cytokines and several other ECM regulating materials [27], [28], [29].

In the present study, we found that conditioned medium of UCB-MSCs contained a variety of growth factors such as EGF, bFGF, TGF-b, PDGF, HGF, Collagen type 1 and exhibited the most prominent effect in migration and proliferation of HDFs compared to other sources of hMSC-CM. USC-CM treatment increased the production of Collagen type 1, Collagen type 3, Fibronectin and Elastin in the HDFs, which makes it the key cell responsible for collagen production. The major collagenous components of dermis are Collagen type 1 and 3 [30] and collagen remodeling plays an important role in facial rejuvenation.

We found USC-CM contained one of the rejuvenation factors, GDF-11. The expression of GDF-11 was found in the blood of young animals. It declines with aging, and reverses aging in multiple tissues including central nervous system [21], [31], [32]. However, the effects of GDF-11 on HDFs have not yet been addressed previously. In this paper, we discovered for the first time that GDF-11 stimulates growth and secretion of ECM proteins including Collagen type 1 and 3, Elastin and Fibronectin in HDFs. These findings suggest that rejuvenating effect of GDF-11 could be expended to human skin in addition to the specific organs previously reported.

USC-CM can stimulate the productions of ECM such as Collagen, Fibronectin and Elastin, from HDFs and one of these functions of USC-CM is responsible for GDF-11 production and secretion. UCB-MSC produced the highest amount of GDF-11 compared to AD-MSC and BM-MSC. In this paper, we showed that GDF-11 could solely stimulate ECM production from HDFs and HDFs migration. However, there are other undefined factors in USC-CM that can contribute in accelerating the ECM production of HDFs. These other factors must be identified and studied in the near future to confirm the sole effect of GDF-11.

Skin wound repair by BM-MSC was shown in normal and diabetic mice by comparing their allogeneic neonatal dermal fibroblasts [33]. Thus we investigated that USC-CM can accelerate wound healing effect in animal studies by stimulating fibroblast migration (data not shown). Furthermore, our USC-CM treatments are expected to inhibit the synthesis of melanin and the activity of tyrosinase in melanoma B16 cells as AD-MSC-CM do [34].

Research of our previous study found that high amount of EGF including various growth factors exists in USC-CM as exosome forms [35]. Major cytokines, such as EGF, secreted in exosomes are easily integrated to skin tissues since exosomes and cell membranes are common lipid membranes. These are one of the explanations why USC-CM contained cosmetics are effective to human skin. Our USC-CM is cryo-dehydrated powder and mixed with cosmetic base before use. Stability test results showed this mixture is stable for a week in refrigerated condition (data not shown). Lemestr et al. showed that GDF-11 decreased fibroblast senescence [36]. However, it is still unclear if GDF-11 can enhance skin regeneration *via* human applied. In this study, we focused on the effect of conditioned media from human umbilical cord blood-derived mesenchymal stem cells in human skin. *In vivo* tests, every volunteer signed this refrigerated storage condition before application. Even though USC-CM and cosmetic base mixture are treated topically, they can penetrate to deep skin since effective factors of USC-CM are encapsulated with exosomes preserved before usage and they can easily integrate to deep skin tissues.

Taken together, GDF-11 secreted from hUCB-MSCs could stimulate cellular growth and expression of ECM proteins in HDFs. Moreover, topical treatment of USC-CM could decrease skin wrinkles *in vivo* study. These findings suggest that the rejuvenating functions of GDF-11 can be expanded to the skin in addition to the central nervous system.

## References

[1] Braverman I.M., Fonferko E. (1982). Studies in cutaneous aging .1. The elastic fiber network. J. Investig. Dermatol..

[2] Tundis R L.M., Bonesi M., Menichini F. (2015). Potential role of natural compounds against skin aging. Curr. Med. Chem..

[3] Warren R., Gartstein V., Kligman A.M., Montagna W., Allendorf R.A., Ridder G.M. (1991). Age, sunlight, and facial skin - a histologic and quantitative study. J. Am. Acad. Dermatol..

[4] Fisher G.J., Kang S.W., Varani J., Bata-Csorgo Z., Wan Y.S., Datta S., Voorhees J.J. (2002). Mechanisms of photoaging and chronological skin aging. Arch. Dermatol..

[5] Rittie L., Fisher G.J. (2002). UV-light-induced signal cascades and skin aging. Ageing Res. Rev..

[6] Fulop T., Khalil A., Larbi A. (2012). The role of elastin peptides in modulating the immune response in aging and age-related diseases. Pathol. Biol..

[7] Maity N., Nema N.K., Abedy M.K., Sarkar B.K., Mukherjee P.K. (2011). Exploring Tagetes erecta Linn flower for the elastase, hyaluronidase and MMP-1 inhibitory activity. J. Ethnopharmacol..

[8] Udompataikul P.Sa.P.P.M. (2009). An oral nutraceutical containing antioxidants, minerals and glycosaminoglycans improves skin roughness and fine wrinkles. Int. J. Cosmet. Sci..

[9] Seo J.Y., Chung J.H. (2006). Thermal aging: a new concept of skin aging. J. Dermatol. Sci..

[10] Jin H.J., Bae Y.K., Kim M., Kwon S.J., Jeon H.B., Choi S.J., Kim S.W., Yang Y.S., Oh W., Chang J.W. (2013). Comparative analysis of human mesenchymal stem cells from bone marrow, adipose tissue, and umbilical cord blood as sources of cell therapy. Int. J. Mol. Sci..

[11] Kern S., Eichler H., Stoeve J., Kluter H., Bieback K. (2006). Comparative analysis of mesenchymal stem cells from bone marrow, umbilical cord blood, or adipose tissue. Stem Cells.

[12] Ding D.C., Chang Y.H., Shyu W.C., Lin S.Z. (2015). Human umbilical cord mesenchymal stem cells: a new era for stem cell therapy. Cell Transplant..

[13] Rivers J.K. (2014). Stem cells for skin rejuvenation: are we there yet?. J. Cutan. Med. Surg..

[14] Kim W.S., Park B.S., Sung J.H., Yang J.M., Park S.B., Kwak S.J., Park J.S. (2007). Wound heating effect of adipose-derived stem cells: a critical role of secretory factors on human dermal fibroblasts. J. Dermatol. Sci..

[15] Sasaki M., Abe R., Fujita Y., Ando S., Inokuma D., Shimizu H. (2008). Mesenchymal stem cells are recruited into wounded skin and contribute to wound repair by transdifferentiation into multiple skin cell type. J. Immunol..

[16] Kim W.S., Park B.S., Park S.H., Kim H.K., Sung J.H. (2009). Antiwrinkle effect of adipose-derived stem cell: activation of dermal fibroblast by secretory factors. J. Dermatol. Sci..

[17] Yoon B.S., Moon J.H., Jun E.K., Kim J., Maeng I., Kim J.S., Lee J.H., Baik C.S., Kim A., Cho K.S., Lee J.H., Lee H.H., Whang K.Y., You S. (2010). Secretory profiles and wound healing effects of human amniotic fluid-derived mesenchymal stem cells. Stem Cells Dev..

[18] Fabi S., Sundaram H. (2014). The potential of topical and injectable growth factors and cytokines for skin rejuvenation. Facial Plast. Surg..

[19] McPherron A.C., Lawler A.M., Lee S.J. (1999). Regulation of anterior posterior patterning of the axial skeleton by growth differentiation factor 11. Nat. Genet..

[20] Kaiser J. (2014). Aging 'rejuvenation factor' in blood turns back the clock in old mice. Science.

[21] Katsimpardi L., Litterman N.K., Schein P.A., Miller C.M., Loffredo F.S., Wojtkiewicz G.R., Chen J.W., Lee R.T., Wagers A.J., Rubin L.L. (2014). Vascular and neurogenic rejuvenation of the aging mouse brain by young systemic factors. Science.

[22] Battaglia T.C. (2005). GDF-5 deficiency alters stress-relaxation properties in mouse skin. J. Dermatol. Sci..

[23] Seo S.R.L.K.W., Bhandari D.R., Roh K.H., Park S.B., So A.Y., Jung J.W., Seo M.S., Kang S.K., Lee Y.S., Kang K.S. (2009). OCT4A contributes to the stemness and multi-potency of human umbilical cord blood-derived multipotent stem cells (hUCB-MSCs). Biochem. Biophys. Res. Commun..

[24] Flynn A., Barry F., O'Brien T. (2007). UC blood-derived mesenchyrnal stromal cells: an overview. Cytotherapy.

[25] Secco M., Zucconi E., Vieira N.M., Fogaca L.L.Q., Cerqueira A., Carvalho M.D.F., Jazedje T., Okamoto O.K., Muotri A.R., Zatz M. (2008). Multipotent stem cells from umbilical cord: cord is richer than blood!. Stem Cells.

[26] Pillouer-Prost A.L. (2003). Fibroblasts: what's new in cellular biology?. J. Cosmet. Laser Ther..

[27] Lozito T.P., Jackson W.M., Nesti L.J., Tuan R.S. (2014). Human mesenchymal stem cells generate a distinct pericellular zone of MMP activities via binding of MMPs and secretion of high levels of TIMPs. Matrix Biol..

[28] Tamama K., Kerpedjieva S.S. (2012). Acceleration of wound healing by multiple growth factors and cytokines secreted from multipotential stromal cells/mesenchymal stem cells. Adv. Wound Care.

[29] Zhukareva V., Obrocka M., Houle J.D., Fischer I., Neuhuber B. (2010). Secretion profile of human bone marrow stromal cells: donor variability and response to inflammatory stimuli. Cytokine.

[30] Garrone R., Lethias C., LeGuellec D. (1997). Distribution of minor collagens during skin development. Microsc. Res. Tech..

[31] Conboy I.M., Conboy M.J., Wagers A.J., Girma E.R., Weissman I.L., Rando T.A. (2005). Rejuvenation of aged progenitor cells by exposure to a young systemic environment. Nature.

[32] Ruckh J.M., Zhao J.W., Shadrach J.L., van Wijngaarden P., Rao T.N., Wagers A.J., Franklin R.J.M. (2012). Rejuvenation of regeneration in the aging central nervous system. Cell Stem Cell.

[33] Wu Y.J., Chen L., Scott P.G., Tredget E.E. (2007). Mesenchymal stem cells enhance wound healing through differentiation and angiogenesis. Stem Cells.

[34] Kim W.S., Park S.H., Ahn S.J., Kim H.K., Park J.S., Lee G.Y., Kim K.J., Whang K.K., Kang S.H., Park B.S., Sung J.H. (2008). Whitening effect of adipose-derived stem cells: a critical role of TGF-beta 1. Biol. Pharm. Bull..

[35] Kim Y.J., Yoo S.M., Park H.H., Lim H.J., Kim Y.L., Lee S., Seo K.W., Kang K.S. (2017). Exosomes derived from human umbilical cord blood mesenchymal stem cells stimulates rejuvenation of human skin. Biochem. Biophys. Res. Commun..

[36] Lemestr A., Hajem N., Choulot J., Bonnans M., Serre C., Restellini L., Botto J., Chabert R., Cucumel K. (2018). Growth differentiation factor 11 (GDF11) modulation in skin rejuvenation. J. Investig. Dermatol..

