# Supplementary material for: Conditioned media from human umbilical cord blood-derived mesenchymal stem cells stimulate rejuvenation function in human skin
Source: Biochem Biophys Rep. 2018 Oct 25;16:96–102. doi: 10.1016/j.bbrep.2018.10.007 (PMC6205340; doi:10.1016/j.bbrep.2018.10.007)
Supplement: Supplementary file 1 — Supplementary material [file mmc1.zip › Disclosure of interests.docx]

Disclosure of interests

The authors declare no potential conflicts of interest.
