# Supplementary material for: Conditioned media from human umbilical cord blood-derived mesenchymal stem cells stimulate rejuvenation function in human skin
Source: Biochem Biophys Rep. 2018 Oct 25;16:96–102. doi: 10.1016/j.bbrep.2018.10.007 (PMC6205340; doi:10.1016/j.bbrep.2018.10.007)
Supplement: Supplementary file 2 — Supplementary material [file mmc2.doc]

**Supplementary Tables and Figures**

**Supplementary Table 1. Primers used in research.**

| Gene | Species | Primer |
| --- | --- | --- |
| Collagen type1 | Human | F 5’-cacagaggtttcagtggtttgg-3’ |
|  |  | R 5’-gcaccagtagcaccatcatttc -3’ |
| Collagen type 3 | Human | F 5’-ctgaaattctgccatcctgaac-3’ |
|  |  | R 5’-ggattgccgtagctaaactgaa -3’ |
| MMP-1 | Human | F 5’-ttgagaaagccttccaactctg-3’ |
|  |  | R 5’-ccgcaacacgatgtaagttgta-3’ |
| Fibronectin | Human | F 5’-aagattggagagaagtgggacc -3’ |
|  |  | R 5’-gagcaaatggcaccgagata-3’ |
| Elastin | Human | F 5’-gggttgtgtcaccagaagca-3’ |
|  |  | R 5’-caaccccgtaagtaggaatgc3’ |
| RPL13A | Human | F 5’-gcacgaccttgagggcagcc -3’ |
|  |  | R 5’-catcgtggctaaacaggtactg-3’ |
| GAPDH | Human | F 5’-gagtcaacggatttggtcgt-3’ |
|  |  | R 5’-catcgtggctaaacaggtactg-3’ |

**Supplementary Table 2. Concentration of secreted proteins of USC-CM**

| Secreted proteins | Concentration (pg/ml) |
| --- | --- |
| bFGF | 2634 |
| EGF | 2305.72 |
| Fibronectin | 80 |
| GDF-11 | 50 |
| HGF | 191.42 |
| KGF | 45 |
| PDGF-AA | 192.06 |
| TGF-b1 | 90.25 |
| Collagen type 1 | 4.15 x 10^8^ |
| VEGF | 69.06 |

bFGF: basic fibroblast growth factor; EGF: epidermal growth factor; GDF-11: growth

differentiation factor-11; HGF: hepatocyte growth factor; KGF: keratinocyte growth factor;

PDGF-AA: platelet derived growth factor-AA; TGF-b1: transforming growth factor-beta 1;

USC-CM: human umbilical cord blood-derived mesenchymal stem cell conditioned media;

VEGF: vascular endothelial growth factor.

**Supplementary Table 3. Statistical analysis of dermal density by ultrasonography**

| **Week** | **N** | **Mean ± SEM** | **Increase (%)** | ***p*-value** |
| --- | --- | --- | --- | --- |
| **0** | 22 | 14.20 ± 0.09 | - | - |
| **2** | 22 | 14.33 ± 0.08 | 0.92 | 0.224 |
| **4** | 22 | 14.55 ± 0.10 | 2.46 | 0.004^***^ |

^***^ *P* < 0.005

**Supplementary Table 4. Statistical analysis of skin wrinkle by 3D image**

| **Parameter** | **Week** | **N** | **Mean ± SEM** | **Increase (%)** | ***p*-value** |
| --- | --- | --- | --- | --- | --- |
| **Ra** | 0 | 22 | 13.62 ± 0.56 | - | - |
|  | 2 | 22 | 13.40 ± 0.50 | -1.62 | 0.279 |
|  | 4 | 22 | 13.08 ± 0.45 | -3.96 | 0.126 |
| **Rmax** | 0 | 22 | 94.93 ± 4.31 | - | - |
|  | 2 | 22 | 92.97 ± 3.34 | -2.06 | 0.283 |
|  | 4 | 22 | 90.35 ± 3.44 | -4.82 | 0.049^*^ |
| **Rz** | 0 | 22 | 68.87 ± 2.49 | - | - |
|  | 2 | 22 | 67.10 ± 2.08 | -2.57 | 0.053 |
|  | 4 | 22 | 66.04 ± 2.15 | -4.11 | 0.060 |
| **Rp** | 0 | 22 | 42.66 ± 1.19 | - | - |
|  | 2 | 22 | 41.70 ± 1.20 | -1.49 | 0.402 |
|  | 4 | 22 | 40.35 ± 1.02 | -4.68 | 0.007^**^ |
| **Rv** | 0 | 22 | 57.23 ± 3.43 | - | - |
|  | 2 | 22 | 56.00 ± 2.54 | -2.15 | 0.417 |
|  | 4 | 22 | 54.59 ± 2.70 | -4.61 | 0.147 |

Ra: Arithmetic average value of profile peaks within the total measuring length; Rmax: Maximum of all peak-to-valley values Rt, measured over the assessment length; Rz: Average maximum height of the profile; Rp: Maximum profile peak height; Rv: Maximum profile valley height ; ^*^ *P* < 0.05, ^**^ *P* < 0.01

**Supplementary Fig. 1.** Comparative analysis of USC-CM, AD-MSC-CM and BM-MSC-CM.

(A) Human growth factor antibody array analysis of USC-CM, AD-MSC-CM and BM-MSC-CM. (B) Densitometric analysis of proteins associated with skin rejuvenation.


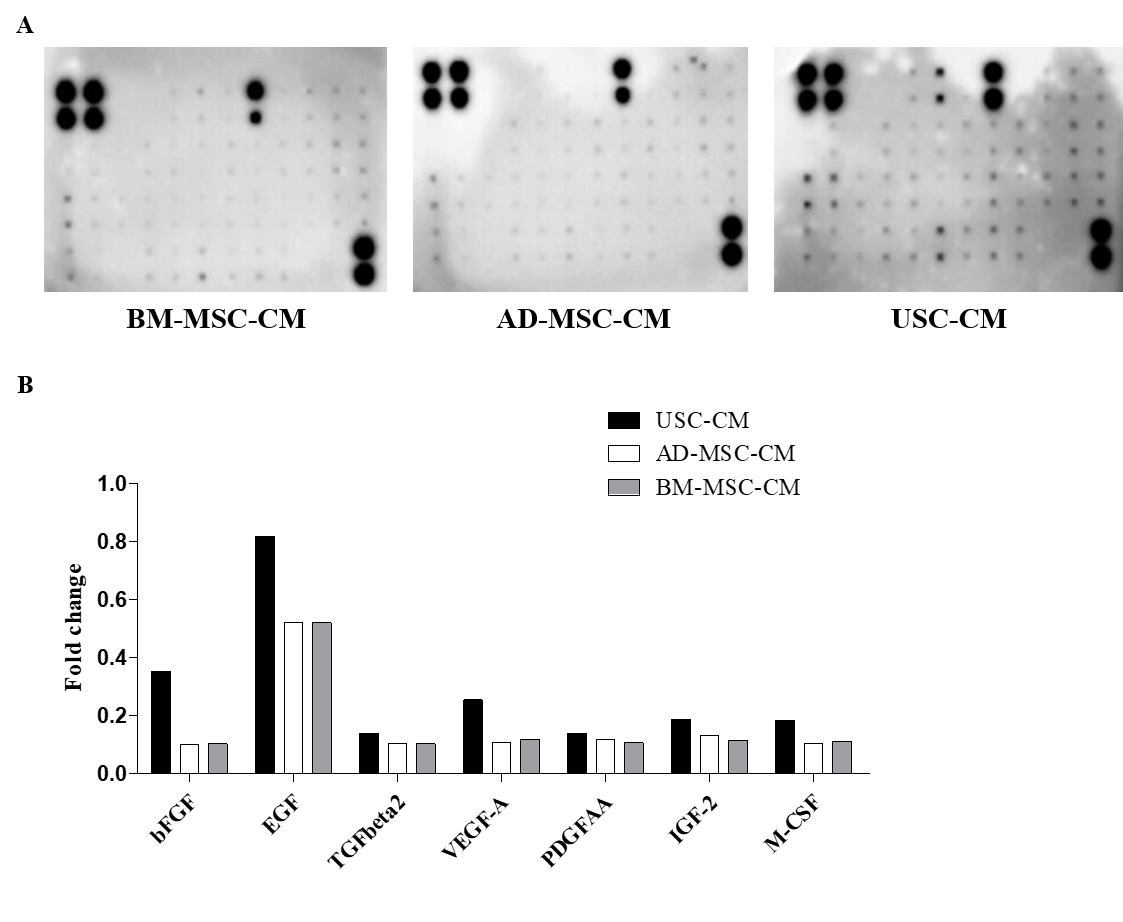


**Supplementary Fig. 2.** *In vivo* tests of USC-CM contained cosmetics.

(A) Dermal density measurement after daily USC-CM contained cosmetics treatment. Density was increased following time course. (B) Decreased skin wrinkle of eye end area after daily USC-CM contained cosmetics treatment.

**
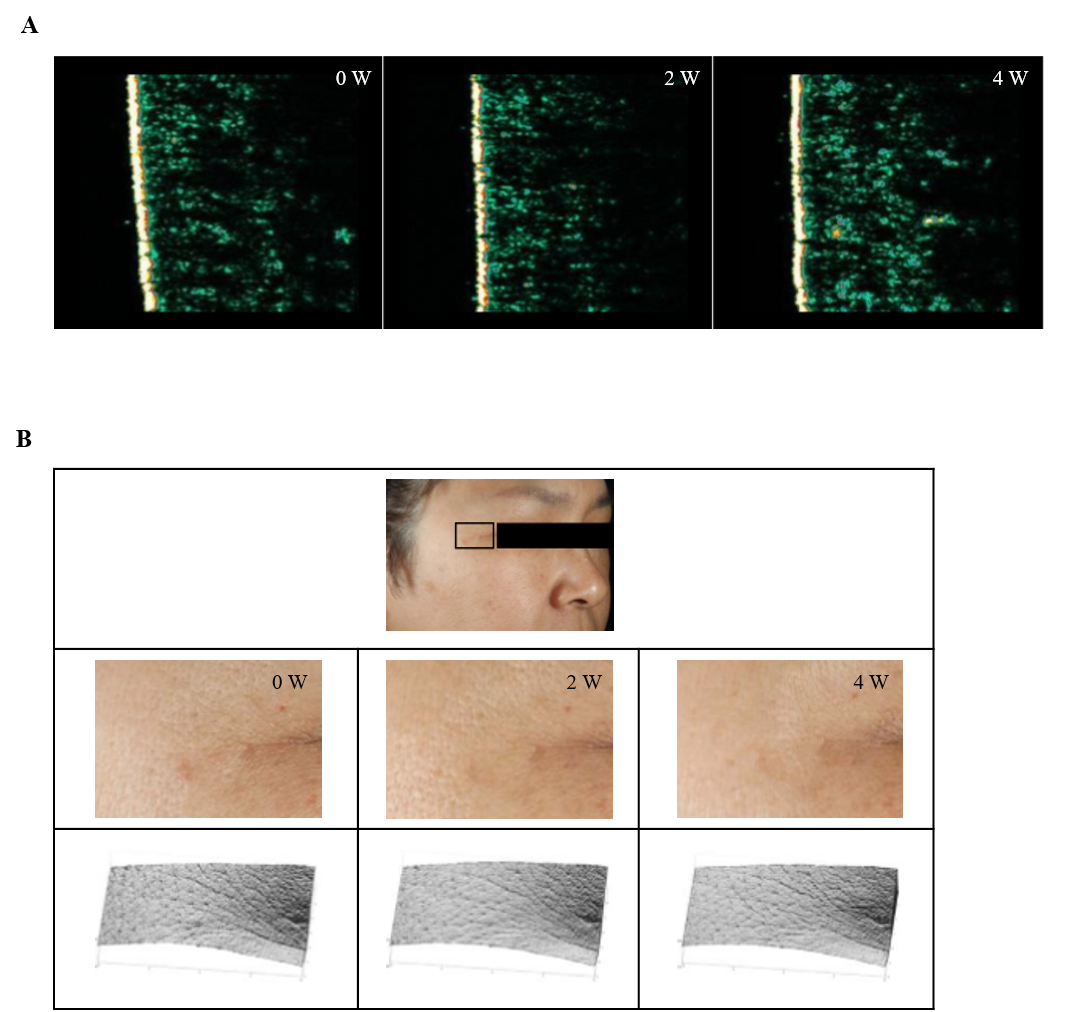
**

**Supplementary Fig. 3.** GDF-11 activates SMAD2 and SMAD3 signals in HDFs.

(A) GDF-11 induced an increase of p-SMAD2 and p-SMAD3 expression in HDFs.

**
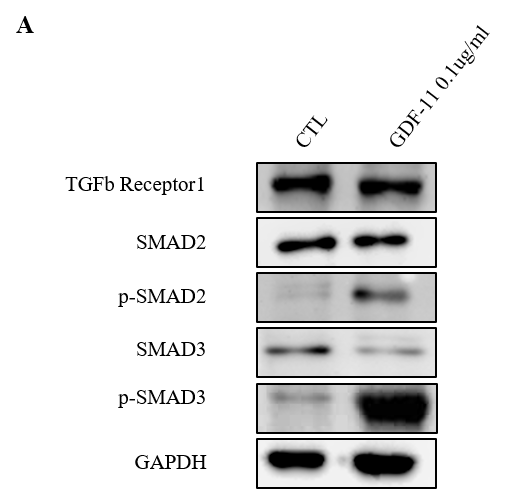
**
