# Supplementary material for: Conditioned media from human umbilical cord blood-derived mesenchymal stem cells stimulate rejuvenation function in human skin
Source: Biochem Biophys Rep. 2018 Oct 25;16:96–102. doi: 10.1016/j.bbrep.2018.10.007 (PMC6205340; doi:10.1016/j.bbrep.2018.10.007)
Supplement: Supplementary file 3 — Supplementary material [file mmc3.doc]

**Supplementary Materials and Methods**

**Human Growth Factor Antibody Array**

Human growth factors were analyzed by using a Human Growth Factor Antibody Array (Cat. No. AAH-GF-1-8, RayBiotech) according to the manufacturer’s instructions. Briefly, after adjusting the membranes into the incubation chamber, the membranes were blocked by adding 2 ml blocking buffer to each incubation well for 30 min at room temperature. Then, the blocking buffer was discarded and samples (BM-MSC-CM, AD-MSC-CM and USC-CM; 2 ml) were added to the wells of the membranes for 2 h at room temperature. After that period, samples were discarded and the membranes were washed 3 times for 5 min with 2 ml wash buffer I and twice for 5 min with 2 ml wash buffer II. The biotin-conjugated antibody solution was added to each well of the membranes and incubated for 2 h at room temperature. Again, the membranes were extensively washed with wash buffers I and II. Subsequently, the 1X HRP-streptavidin solution was added to each well and incubated for 2 h at room temperature. After additional washing steps with wash buffers I and II, the membranes were removed from the incubation frame. Membranes were developed using detection buffer and quantified using a densitometer. Mean pixel density was quantified using Image J analysis. Signal intensity was normalized to internal positive controls for comparison).

***In vivo* human test**

*In vivo* tests on human were performed by Dermapro (Seoul, Korea). Twenty four females were tested and each subject was informed with directions before application (IRB, 1-220797-A-N-02-DICN15070). Evaluation of skin wrinkles, color, and dermal density was performed before application of the product, 2 and 4 weeks after application. Skin wrinkles were measured using PRIMOS**®**premium (GFMesstechnik GmbH, Germany). Skin color was measured using Spectrophotometer**®**CM-2500d (Minolta, Tokyo, Japan). Dermal density was measured using Dermascan**®**C (Cortex, Hadsund, Denmark).
